# Physiological and transcriptomic analyses revealed the change of main flavor substance of *Zygosaccharomyces rouxii* under salt treatment

**DOI:** 10.3389/fnut.2022.990380

**Published:** 2022-08-24

**Authors:** Rongqiang Pei, Gongbo Lv, Binrong Guo, Yuan Li, Mingqiang Ai, Bin He, Runlan Wan

**Affiliations:** ^1^Jiangxi Key Laboratory of Bioprocess Engineering, College of Life Sciences, Jiangxi Science and Technology Normal University, Nanchang, China; ^2^Department of Oncology, The Affiliated Hospital of Southwest Medical University, Luzhou, China

**Keywords:** *Zygosaccharomyces rouxii*, salt treatment, transcriptomics, flavor substance, physiological

## Abstract

*Zygosaccharomyces rouxii* was a highly salt-tolerant yeast, playing an important role in soy sauce fermentation. Previous studies reported that *Z. rouxii* under salt treatment produces better fermented food. However, the detailed change of main flavor substance was not clear. In this study, the physiological and transcriptomic analyses of *Z. rouxii* under salt treatment was investigated. The results revealed the high salt tolerance of *Z. rouxii*. Analysis of physiological data showed that the proportion of unsaturated fatty acids was significantly increased with the increment of salt concentrations. The analysis of organic acids showed that the content of succinic acid was significantly higher in the salt-treated *Z. rouxii* while oxalic acid was only identified at the 18% salt concentration-treated group. Results of volatile substances analysis showed that concentrations of 3-methyl-1-butanol and phenylethyl alcohol were significantly increased with the increment of salt concentrations. A comparison of transcriptome data showed that the genes involved in the TCA cycle and the linoleic acid synthesis process exhibited different expressions, which is consistent with the results of physiological data. This study helps to understand the change of main flavor substance of *Z. rouxii* under salt treatment and guide their applications in the high salt liquid state fermentation of the soy sauce.

## Introduction

*Zygosaccharomyces rouxii* belongs to the genus *Zygosaccharomyces*, which plays an important part in the manufacture of traditional fermented foods, such as soy sauce and miso. It mainly participates in consuming the sugars and amino acids of the soybeans and wheat during soy sauce fermentation. Apart from catalyzing the glucose conversion into ethanol, *Z. rouxii* is also capable of releasing higher alcohols from free amino acids *via* the Ehrlich pathway and produces a sweet and caramel type of flavor (1). The main flavor substances are two 4-hydroxyfuranones derivatives, 4-hydroxy-2,5-dimethyl-3(2H)-furanone (HDMF) and 4-hydroxy-2(or 5)-ethyl-5(or 2)-methyl-3(2H)-furanone (HEMF), the latter is also a potent antioxidant and has anticarcinogenic effects (2–4). In addition, it was also reported that the quality of fermented foods was improved and the fermentation process could be accelerated by the addition of *Z. rouxii* as a starter culture (5).

The volatile and non-volatile substances, such as organic acids, alcohols, and amino acids, contribute to the nutrition, unique taste, and aroma of fermented foods. Organic acids are one of the crucial compounds present in fermented products that not only have a huge effect on the sour taste but several of which also have other flavor attributes including a bitter or salty taste (6). Additionally, organic acids also affect the pH of the fermented products, which further affects the product quality, such as flavor stability (7, 8). Succinic acid, derived from the fermentation of glucose, is generally found in various fermented foods such as soy sauce, seasonings, and miso. All of these fermentation products have very distinct and marked flavors, which might attribute in part to a flavor enhancement by the small amounts of succinic acid. This would imply that succinic acid may have vital effects on various flavors that cannot be replicated with other food organic acids (9). As a major inhibitory and volatile compound, acetic acid can cause intracellular acidification or cell death and inhibit cell metabolism that acts to stress microbiota, finally leading to the reduction of yeast fermentative performance (10, 11). Intriguingly, acetic acid is generally used in foods as a flavor enhancer and flavoring agent. In addition to ethanol, alcohols, and organic acids, aldehydes, the main aroma-active compounds, ketones, esters, furans, phenols, and pyrazines were known as critical characteristic flavor substances of fermented foods produced *via Z. rouxii* (12).

*Zygosaccharomyces rouxii* inevitably suffer from multiple abiotic stresses covering osmotic, acidic, oxidative, temperature, and alcohol stresses during the process of fermented food production. Especially the high salt concentration triggers osmotic stress resulting in structural and physiological damage of cells, and thus negatively affects the growth and survival of organisms (13, 14). Previous studies have reported that *Z. rouxii* harbor a good tolerance toward high concentrations of salt, and the related physiological, transcriptomic, and metabolomics analysis of the salt tolerance mechanism of *Z. rouxii* have been comprehensively elucidated (5, 15, 16). Even though the high salinity environment leads to the injury cell and the low metabolic activity of *Z. rouxii*, on the other hand, a characteristic of *Z. rouxii* fermentation under that condition was also observed in the past several decades, accelerating and enhancing the flavor formation of fermented foods (17, 18). In addition, *Z. rouxii* is capable of slowly fermenting the sugars and amino acids present in high salty, typically in 18–20% NaCl environment, releasing flavors vital to the quality during soy sauce fermentation (19). Considerable investigations have been made regarding the enhancing and accelerating flavor formation of fermented foods by *Z. rouxii* under the high salinity environment in the last several decades. Nevertheless, to the best of our knowledge, little progress has been made on the detailed mechanism of enhancing and accelerating flavor formation by *Z. rouxii* under the above-mentioned abiotic stress. In the post-genomic era, with the advance of the omics approaches such as metabolomics, genomics, transcriptomics, and proteomics for flavor materials component and ratio analyses, a comprehensive understanding of the mechanisms, processes and environmental stress responses might obtain about the functions of *Z. rouxii* on fermented foods (15, 16, 20). These may also help to understand the genetic, biochemical, and physiological constraints on the metabolic processes of *Z. rouxii* in a high salinity environment.

High-salt condition is the main environmental factor in the high salt liquid state fermentation of the soy sauce (18–20% salt concentration), and *Z. Rouxii* would inevitably be subject to high-salinity stress. The present study aims to delineate the related mechanism of flavor formation by *Z. rouxii* under a high salinity environment. Based on physiological and transcriptome analysis, the salt tolerance response of *Z. rouxii* including growth performance, changes in organic acids content, intracellular fatty acid, and volatile substances, as well as the overall transcription levels of key genes were investigated. Results of this study not only contribute to further completing the mechanism of flavor formation of *Z. rouxii* under high salt stress, but also might be beneficial to enhance and accelerate flavor formation, and thus ultimately improve its fermentation characteristics and guide their applications in the production of traditional fermented foods.

## Materials and methods

### Fungal strain and cultivation

The *Z. rouxii* CICC 32899 strain, purchased from China Center of Industrial Culture Collection, was used in this study. It was grown statically in a YPD medium (yeast paste 10 g/L, protein 20 g/L, glucose 20 g/L, pH 6.0) at 30°C for 24 h for activating strain. To observe the growth of *Z. rouxii* at different salt concentrations, the activated *Z. rouxii* was inoculated in an YPD medium treated with different salt concentrations (0, 6, 12, and 18%) for 3 days, each sample was performed in triplicate.

### Determination of OD values in *Zygosaccharomyces rouxii* under salt stress

The growth of *Z. rouxii* was mainly reflected by the OD values of *Z. rouxii*. *Z. rouxii* had a maximum absorption at 600 nm, thus this wavelength was chosen to determine the OD values. All samples were collected for 12, 24, 48, 60, and 72 h, and then the bacterial broth was diluted in a ratio of 1:3 for determination the OD values. It was determined by the UV spectrophotometer with a wavelength of 600 nm, deionized water as a control and the diluted bacteria solution as samples. The OD values are equal to the OD read multiplied by the dilution multiple. Each sample was performed in triplicate.

### Determination of intracellular fatty acid and volatile substances

Lipids were extracted from cell homogenates and methylated by following previously described methods (21). Specifically, *Z. rouxii* mycelia were filtered, collected, washed with distilled water, and freeze dried. After that, the mycelia of each group were powdered and weighed. The same weight of mycelia powder was subjected to lipid extraction. The lipid extracts were incubated in chloroform with 2% H_2_SO_4_–MeOH solution at 70°C for 2 h to obtain fatty acid methyl esters (FAMEs). The FAME components were separated and analyzed by QP2010 gas chromatography-mass spectrometry (GC-MS) (Shimazjin, Kyoto, Japan). The system was equipped with Supelco SP-2340 fused silica capillary column (30 m × 0.25 mm i.d., with a film thickness of 0.2 μm; Bellefonte, PA, United States). FAMEs were identified by comparing their mass spectra with a spectrum database. FA peaks were identified based on comparisons of their retention times to the external standards or similarity search. The relative amounts of individual FA components were performed by using the peak area of the most intensive ion of each peak, and the percentage of UFAs was also calculated (21).

Same treated samples were also used to determine the volatile substances *via* GC-MS based on Benucci et al. method with some changes (22). It analyzed by QP2010 gas chromatography-mass spectrometry (GC-MS) (Shimazjin, Kyoto, Japan). The system was equipped with Supelco SP-2340 fused silica capillary column (30 m × 0.25 mm i.d., with a film thickness of 0.25 μm; Bellefonte, PA, United States).

### Determination of organic acids content

After 3 days of liquid culture, *Z. rouxii* mycelium was filtered with a 0.45 μm membrane, and then the content of organic acids was determined for the following analysis. Briefly, the mycelia were lyophilized, vacuum freeze dried to a constant weight, and ground to powder. Two grams of dry powder were weighted from each sample was used to perform High Performance Liquid Chromatography (HPLC) detection. HPLC assay was conducted on Waters Alliance e2695 HPLC (Milford, MA, United States) using a UV detector set at 210 nm equipped with Aminex HPX-87H (Bio-Rad, CA, United States) column (300 × 7.8 mm, 9 μm). The analysis conditions were as follows: the mobile phase was 0.065% H_3_PO_4_ with isocratic elution at flow rate of 0.6 ml min^–1^, and 10 μl injection sample volume. A standard organic acids curve was generated using tartaric acid, propanedioic acid, acetic acid, citric acid, succinic acid, malic acid, and oxalic acid standard (Sigma-Aldrich, Burlington, MA, United States). These yield of organic acids was calculated using the detected peak area according to the standard curve.

### Preparation of cDNA libraries and RNA sequencing

After obtaining the cell samples, RNA extraction was performed using the fungal RNA kit (Omega Bio-Tek, Norcross, GA, United States). The RNA concentrations were detected by using a NanoDrop ND-1000 spectrophotometer (Thermo Scientific, Wilmington, DE, United States), and the RNA integrity values were analyzed with a Bioanalyzer 2100 (Agilent Technologies, Palo Alto, CA, United States). To ensure reliability and reproducibility, equal quantities of RNA of each pool from three individual cultures were used for cDNA library construction. Afterward, the mRNA was enriched from pooled total RNA using oligo (dT) magnetic beads and then digested into short pieces with fragmentation buffer at 94°C for 5 min and then reversely transcripted into cDNA using random hexamer primers. Using these mRNA fragments as templates, the first-strand cDNA was synthesized, followed by second-strand cDNA synthesis by DNA polymerase I and RNase H (23). The cDNA fragments were purified using QIAquick PCR extraction kit, end repaired, and ligated to Illumina sequencing adapters to create the cDNA library. After that the libraries were sequenced by the Illumina HiSeq 2500 platform (Illumina, San Diego, CA, United States). The obtained raw reads containing adapters or low-quality bases will be further filtered by our previous criterion to get clean reads, and thus, removed (21). Moreover, the Bowtie2 software was used to remove reads that mapped to ribosome RNA (rRNA) database to get the final clean reads, which were further employed for assembly and transcriptome analysis (24). RNA-seq data of *Z. rouxii* under distinct salt stress were deposited in the NCBI/SRA database, under the BioProject accession number PRJNA837122.

### Identification and KEGG enrichment of differentially expressed genes

The obtained clean-read datasets were aligned to *Z. rouxii* reference genome using HISAT2 (25). The RSEM software was used to quantify gene abundances, and the quantification of gene expression level was normalized using the FPKM (Fragments Per Kilobase of transcript perMillion mapped reads) method (26, 27). Differentially expressed genes (DEGs) across samples were identified using DESeq2 v1.18.1 on R package (version 3.4.2). The log_2_ (fold change) over 1 and false discovery rate (FDR) within 0.05 were set as the threshold for significant DEGs (28). Besides, the identified DEGs were carried out into hierarchical clustering, with the KEGG pathway enrichment analysis (29).

### Quantitative real-time PCR analysis

To validate the transcriptional level results from RNA-seq data analysis, six genes including elongase 1–4, D9D, and D12D genes which are involved in the linoleic acid biosynthesis in *Z. rouxii*, were selected for real-time RT-PCR validation. Total RNA was extracted with E.Z.N.A. Fungal RNA Kit (Omega Bio-Tek, Norcross, GA, United States) according to the protocols of the manufacturer. Real-time RT-PCR was performed according to our previous work (30). GAPDH served as the reference gene for the normalization of the target gene expression and to correct for variation between samples. Primers used for the candidate genes were designed based on the Illumina sequencing data by using Primer Premier 5 and listed in Supplementary Table 1. The comparative 2^–ΔΔCT^ method was employed to calculate the relative expression between the target genes.

### Statistical analysis

All the data obtained in this experiment are presented as the mean of three replicates. Data from the same period were evaluated by two-way nested analysis of variance (ANOVA), followed by the least significant difference test (LSD) for mean comparison. All statistical analysis was performed with SAS 9.20 software (SAS Institute Inc., Cary, NC, United States) at a *p* < 0.05.

## Results

### Effect of salt stress on the growth performance of *Zygosaccharomyces rouxii*

**FIGURE 1:**
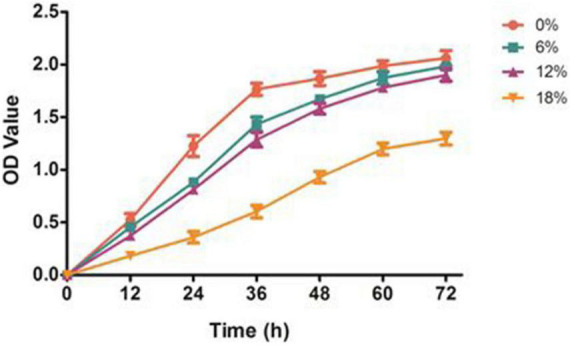
Effect of salt stress on the growth of *Z. rouxii*. 0, 6, 12, and 18% represent different salt concentrations, respectively.

To investigate the effects of salt stress on the growth performance of *Z. rouxii*, a preliminary study of different concentrations of salt treatments was conducted (Figure 1). *Z. rouxii* grew well in the control condition, and the maximum biomass yield (OD 600) reached to 2.1. With the salt concentrations increasing from 0 to 18%, the growth of *Z. rouxii* was gradually inhibited, and the highest OD 600 decreased from 2.1 to 1.2. This result suggested a great effect of salt stress on the growth performance of *Z. rouxii*. Of note, the similar growth performance of *Z. rouxii* was detected at 6 and 12% salt concentration, which illustrated both of these salt concentrations harbor the analogous effect on *Z. rouxii* growth. Moreover, the almost same maximum OD 600 was identified at 0, 6, and 12% salt concentrations, elucidating the high salt tolerance of *Z. rouxii*. The *Z. rouxii* grew slowly and almost stagnant during the period from 60 to 72 h, the samples thus cultured for 72 h were selected for the follow-up analysis.

### Analysis of intracellular fatty acid of *Zygosaccharomyces rouxii* under salt stress

**FIGURE 2:**
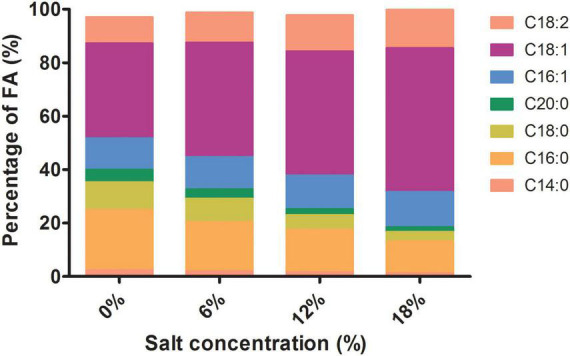
The main intracellular fatty acids content in *Z. rouxii* under salt stress.

Previous studies have documented that changes of the membrane lipid composition have impacts on the membrane fluidity and then affect the stress resistance of yeast (31, 32). *Z. rouxii* inevitably encountered salt stress in the manufacture of high salty foods. However, the effect of salt stress on the fermented food produced by *Z. rouxii* is poorly understood. In the present study, the fatty acid content profiles of *Z. rouxii* grown under salt stress and control conditions were qualitative and quantitative determinations by GC-MS. Our results revealed that the unsaturation of *Z. rouxii* increases with increasing salt concentrations (Supplementary Figure 1). In addition, the main composition of unsaturated and saturated fatty acid was also present on the Figure 2. The composition of fatty acid in *Z. rouxii* cells was dominated by C18 and C16, of them, the proportion of oleic acid (C18:1) was significantly increased with the increment of salt concentrations. Moreover, the proportion of linoleic acid was slightly increased, while palmitoleic acid was hardly increased at different salt concentrations. As for saturated fatty acid, palmitic acid and stearic acid were decreased significantly, while myristic acid and arachidic acid was slightly decreased (Figure 2).

### Analysis of volatile substances of *Zygosaccharomyces rouxii* under salt stress

**FIGURE 3:**
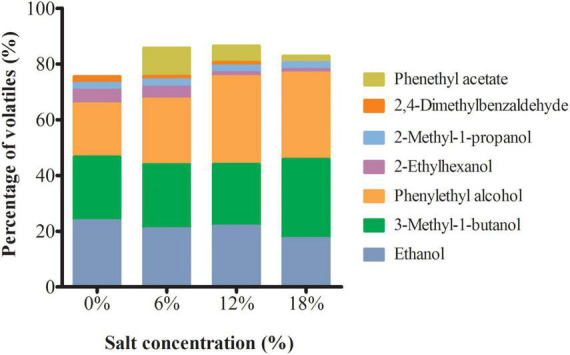
The main volatile substances content in *Z. rouxii* under salt stress.

Volatile substances are essential compositions of flavor in fermented food (33). Here, GC-MS was applied to determine the volatile compounds present in the extracts of *Z. rouxii*. A total of 98, 91, 94, and, 90 volatile compounds was identified at 0, 6, 12, and 18% salt-treated group, of which, 33 volatiles were found commonly to all *Z. rouxii* (Supplementary Table 2). Alcohols and acids made more contributions to the total volatiles in comparison with esters, furans, aldehydes, phenols, and pyrazines. Furthermore, the main seven volatile compounds were identified as the major volatile compounds in *Z. rouxii* due to their higher concentration, such as ethanol, 3-methyl-1-butanol, and phenylethyl alcohol (Figure 3). Among them, the relative contents of ethanol, 2-ethylhexanol, and 2, 4-dimethylbenzaldehyde were decreased with the increment of salt concentration. In contrast, the relative contents of two edible spices, 3-methyl-1-butanol and phenylethyl alcohol were increased, whereas 2-methyl-1-propanol was kept steady under distinct salt stress. Noteworthily, phenethyl acetate, a food flavoring agent, was not detected in the control group.

### Changes in organic acids content of *Zygosaccharomyces rouxii* under salt stress

**FIGURE 4:**
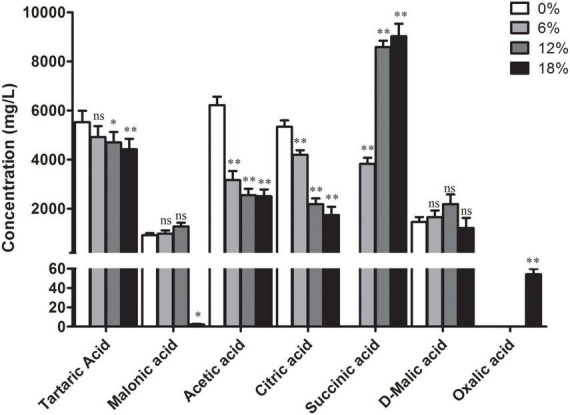
Intracellular content of seven organic acids in *Z. rouxii* under different salt concentrations, 0, 6, 12, and 18% represent different salt concentrations, respectively. The bars represent the average (±SE) of biological repeats. ^ns^No significant difference; *p < 0.05; **p < 0.01.

Yeast bacteria can produce a greater diversity of organic acids during their metabolisms, such as lactic acid, lemon acid, malic acid, and succinic acid (29). Here, the profile of organic acids produced by *Z. rouxii* during fermentation at different salt concentrations was determined. The content of seven main organic acids has distinct degree changes under salt stress compared with the control group (Figure 4). Among them, the content of tartaric acid, acetic acid, and citrate acid was decreased significantly with the increment of salt concentrations. In contrast, no significantly increased content of malonic acid and D-malic acid was identified with the salt treatment, with the exception of 18% salt concentration, of which the content of malonic acid was decreased significantly. In addition, succinic acid was not detected in the control group and its content significantly increased with the increment of salt concentrations while oxalic acid was only identified in the 18% salt concentration-treated group.

### Overview of transcriptome sequencing

To investigate the environmental stress response of *Z. rouxii* induced by salt, a transcriptome analysis based on RNA sequencing of *Z. rouxii* that was subjected to distinct salt concentrations were performed. This resulted into the generation of 43.84, 43.16, 45.05, and 45.66 million clean reads per library, respectively (Table 1). The GC content for all treatments was approximately 43%, and the % ≥Q30 (99.9% accuracy of bases) was greater than 94.4% for all samples, elucidating a good quality of the sequencing data. Moreover, the clean reads were aligned to *Z. rouxii* genome sequence, and more than 83% of the clean reads for each sample were uniquely mapped to the genome. A summary of the RNA-seq sequencing was shown in Table 1.

**TABLE 1:** Summary of the sequencing data of *Z. rouxii* under salt stress.

Samples	Clean reads	GC content	≥Q30	Mapped reads	Unique mapped	Multiple mapped
Control	43,842,750	43.44%	94.48%	39,983,819 (91.20%)	39,273,892 (89.58%)	709,927 (1.62%)
6%	43,164,604	42.61%	94.55%	36,394,985 (84.32%)	35,909,796 (83.19%)	485,189 (1.12%)
12%	45,056,174	43.71%	94.79%	40,484,678 (89.85%)	39,832,104 (88.41%)	652,574 (1.45%)
18%	45,667,748	43.70%	94.54%	41,373,537 (90.60%)	40,587,615 (88.88%)	785,922 (1.72%)

### Differentially expressed genes analysis of *Zygosaccharomyces rouxii* transcriptome under salt concentrations

**FIGURE 5:**
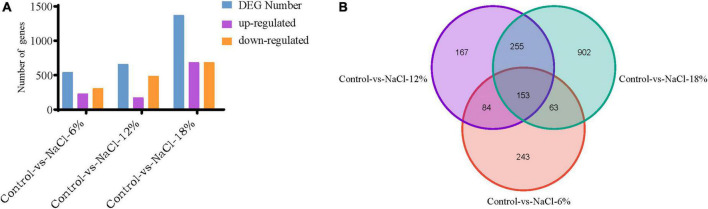
Distribution of differently expressed genes (DEGs) identified in the three samples. **(A)** Distribution of DEGs in the control group as compared to the salt-treated group. **(B)** Venn diagram exhibiting DEG distribution in three samples. NaCl-6%, -12%, and -18% represent the salt concentration of 6, 12 and 18% group, respectively.

To search genes with altered expression levels treated by different salt concentrations, the overall transcription levels of genes was quantified by RPKM metrics. Based on the global transcriptional changes from normalizing the DEG data, a total of 2,575 genes showed altered expression levels in the three salinity treatment groups, as compared to the control. There were 543, 659, and 1,373 DEGs that was identified in the control group and three salinity treatment groups, respectively (Figure 5A). Among them, 232, 173, and 685 genes expression being up-regulated at three salt concentrations compared to control groups, separately, whereas 311, 486, and 688 genes were down-regulated. To better present the effect of salt stress on *Z. rouxii*, a Venn diagram of DEG distribution at these groups was constructed for follow-up analysis due to their significant differences (Figure 5B). It was observed that only 153 DEGs were commonly shared among the distinct salt concentrations. It is noteworthy that the number of specific DEGs between WT-vs-NaCl-18 groups was significantly higher than that of the other two groups, revealing the involvement of complex developmental events of *Z. rouxii* cells under 18% salinity treatments (Figure 5B).

### Differentially expressed genes involved in the citrate cycle

**FIGURE 6:**
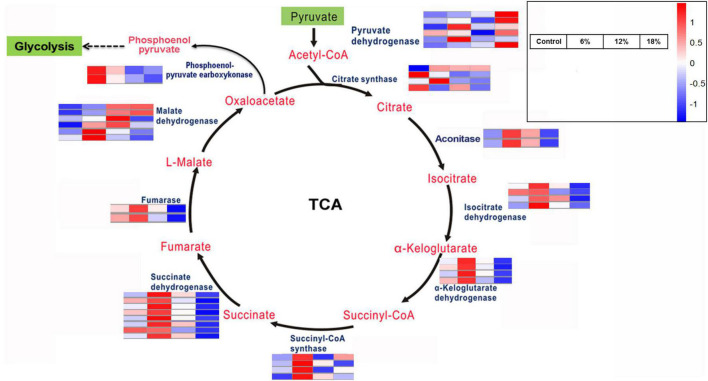
Key enzymes encoded by the DEGs involved in the citrate cycle.

The citrate cycle (TCA cycle, Krebs cycle) is a crucial aerobic pathway involved in for the final steps of the oxidation of carbohydrates and fatty acids. Hence, differentially expressed genes involved in the TCA cycle was investigated according to the KEGG enrichment analysis. A total of 42 DEGs encodings 10 enzymes were identified to be involved in different steps in carbohydrate metabolism (Figure 6). In addition to phosphoenol-pyruvate carboxykinase, fumarase, and aconitase, both of which are encoded by two DEGs, the remaining enzymes responsible for TCA cycle are encoded by four to eight DEGs, such as succinate dehydrogenase (eight DEGs). The expression level of a majority of these DEGs was up-regulated obviously under 6% salt concentration treatment, and only a partly of DEGs was up-regulated at 12%, while the remaining DEGs were down-regulated or harbor no remarkable change. Intriguingly, almost all of the enzyme-encoding genes identified in the TCA cycle was significantly down-regulated under 18% salinity treatment, for example, succinate dehydrogenase and aconitase gene, and with special attention paid to several malate dehydrogenase and pyruvate dehydrogenase gene, which possessed a high expression level.

### Expression analysis of linoleic acid biosynthesis genes under salt stress

**FIGURE 7:**
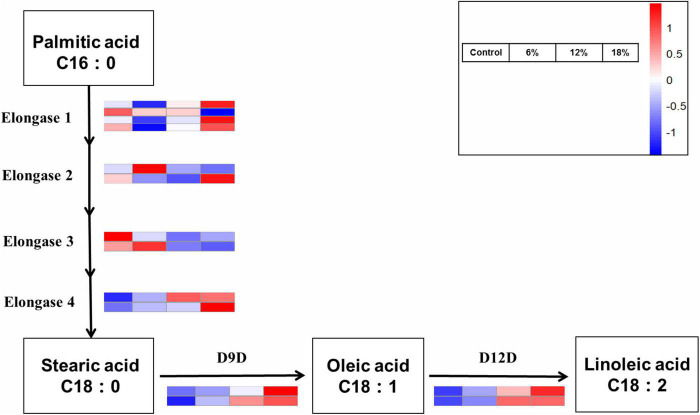
Differentially expressed genes involved in the linoleic acid biosynthesis pathways.

Linoleic acid, a principal essential fatty acid, is also a critical structural component of cell membranes and has effect on cell membrane properties like fluidity, flexibility and permeability. These are six crucial enzymes involved in linoleic acid biosynthesis pathway, including elongase 1–4, two rate-limiting enzymes delta 9 fatty acid desaturases (D9D) and delta 12 fatty acid desaturases (D12D). According to the KEGG pathway analysis, 14 genes responsible for linoleic acid biosynthesis in *Z. rouxii* under salt stress were identified (Figure 7). Just three genes were up-regulated under 6% salinity treatment, the remaining gene were down-regulated or hardly changed, and the same number up-regulated and down-regulated genes were observed under 12% salinity treatment. Additionally, the great majority of genes encoding linoleic acid biosynthesis-related key enzymes were up-regulated under 18% salinity treatment. It has to be noted that four D9D and D12D genes were significant up-regulated under 6 and 18% salinity treatments. This result illustrated that high salt stress is beneficial to unsaturated fatty acid accumulation, which was consistent to the result of the proportion of unsaturated fatty acid determined before. Given that these six genes with notable expression levels in the distinct salt stress, the activity of D9D and D12D genes was enhanced at periods corresponding to linoleic acid production, implying a close correlation between salt stress and transcriptional regulation. This result was further supported by the relative expression levels of gene encoding elongase and D12D using qRT-PCR (Supplementary Figure 2).

## Discussion

Accelerating and enhancing the flavor formation of fermented foods was a paramount pursuit of industrial production. The industrial important microbes, *Z. rouxii*, was observed this characteristic of fermentation under high salinity condition over the past few decades, and related molecular mechanism was being explored. Nevertheless, no convincing evidence or strongly explanation was obtained regarding the related molecular mechanism of accelerating and enhancing the flavor formation by *Z. rouxii* fermentation under high salinity condition. Herein, we performed a physiological and transcriptomic analysis to reveal the main flavor substance of *Z. rouxii* during fermentation under high-salt treatment conditions.

High salinity environment represents a stress induced high osmotic perturbation to fungi cells due to the excess salt disturbs osmotic potential and results in metabolic toxicity. In the present study, the growth performance of *Z. rouxii* was significantly decreased by salt stress, especially in high salt concentration (Figure 1), and this result was analogous to the study of Wang et al. (15). Meanwhile, the number of DEGs reached a peak in the 18% salt treatment group (1,373) while the number was reduced at 6 and 12% salinity (543 and 659), indicating a larger degree of change in gene expressions in the high salt group than that in the low salt treatment (Figure 5). The similar change of gene expressions was also observed in our previous studies on *Aspergillus oryzae* (21). Further more, the characteristic of *Z. rouxii* fermentation under hyper-osmotic condition, accelerating and enhancing the flavor formation of fermented foods, thus require a strong salt tolerance of *Z. rouxii* cells for the long-term fermentation. Previous researches pointed out that several compatible solutes such as carbohydrates, intracellular glycerol, amino acids, and trehalose were accumulated under salt stress, which can be used as osmotic protectants against stress-induced perturbations (15, 34). Aside from the accumulation of these osmotic protectants, changes in the membrane composition and properties represent an essential factor in the adaptation to high salinity environment (35). It is reported that *Z. rouxii* cells responded to the changes in lipid composition in the high NaCl condition, especially the increment of phospholipids with high oleic acid contents and decreases in triacylglycerol and linoleic acid (36). Moreover, this change can also affect the leakage of glycerol, and keep from the influx of sodium ions under that conditions (37). Herein, the unsaturation of fatty acids in *Z. rouxii* has a positive correlation with salt concentrations, and the main composition of unsaturated fatty acids was palmitoleic acid, oleic acid, and linoleic acid (Figure 2 and Supplementary Figure 1). These results suggested that *Z. rouxii* produces more unsaturated fatty acids under salt stress, keeps the structure and function of the cells and helps them adapt to the hypertonic environment. Similar results were also found in *Frankia* strains, *A. oryzae*, and halophilic yeast-like melanized fungi (23, 38, 39). Additionally, the relative expression levels of genes related to linoleic acid biosynthesis in *Z. rouxii* under salt stress further supported the conclusion that salinity stress led to an increase in fatty acid unsaturation to maintain the osmotic balance of cells with this condition. In fact, in the case of exposure of *Z. rouxii* cells to salinity stress, 7 genes encoding elongase 1, 2, and 4 (from C16:0 to C18:0) were up-regulated (Figure 7). However, the content of C16:0 and C18:0 were both decreased under salt stress. According to the expression level of D9D and D12D, we may know that large amounts of C18:0 were biosynthesized into oleic acid and linoleic acid. Noteworthily, the potential health benefits of unsaturated fatty acids have attracted attention recently, and evidence is mounting on the role that several unsaturated fatty acids might play in the primary and secondary prevention of cardiovascular disease (40).

The volatile and non-volatile substances, such as organic acids, alcohols, and amino acids, have a considerable contribution to the main aroma of fermented foods. Through the analysis of volatile substances of *Z. rouxii* under salt stress, it is observed that 3-methyl-1-butanol and phenylethyl alcohol were increased with the increment of salt concentrations (Figure 3). 3-Methyl-1-butanol has apple brandy aroma and spicy flavor and is usually use as food spices, mainly for the preparation of apple and banana flavor. It is reported found in over 230 natural sources including apple, apricot, banana, sour cherry, cheese and so on (41). Phenylethyl alcohol usually existed in rose, carnation, hyacinth, Aleppo pine, orange blossom, and other organisms. It has a pleasant floral odor and used as an additive in cigarettes and also used as a preservative in soaps (42). Additionally, it is observed that phenethyl acetate was specially observed in the salt-treat Z. *rouxii*. Phenethyl acetate occurs in a number of essential oils and is a volatile aroma component of many fruits and alcoholic beverages. Phenylethyl acetate is a colorless liquid with a fine rose scent and a secondary, sweet, honey note. It is used in perfumery as a modifier of phenylethyl alcohol, for example, in rose and lilac compositions. In addition, it is used in a large number of aromas, in keeping with its natural occurrence (43). Hence, we speculate that these volatile substances are the main substance enhancing the flavor formation in *Z. rouxii* fermentation under salinity environment.

Further more, distinct degree changes of organic acids content in *Z. rouxii* was observed under salt treatment (Figure 4). The influence of salinity on the content of organic acids varies according to the microorganism and fermented production. The total levels of organic acids were elevated by salinity during the grapevine fermentation, which increased the production of tartaric and malic acids (44). In the less salt-sensitive cv. Korona, the concentration of total organic acids remained fairly constant under salinity environment. In the salt-sensitive cv. Elsanta, the contents of total organic acids, especially citric acid, increased significantly due to salt stress (45). The majority of organic acids were derived from glucose through the citrate cycle. Transcriptome analysis revealed that the pathway of the citrate cycle was activated corresponding to the content of organic acids, of which a majority of related DEGs were significantly up-regulated under salt treatment (Figure 6). The decrease of some organic acids, such as citric acid, may be due to the more conversion of downstream product. From the results, the content of succinic acid was significant higher in the salt-treated *Z. rouxii* while oxalic acid was only identified at the 18% salt concentration-treated group. Accordingly, we speculate that the content of glucose would be decreased under salt treatment, which need to be confirmed by experiment. Succinic acid is used as a dietary supplement for symptoms related to menopause such as hot flashes and irritability and also used as a flavoring agent for food and beverages (46). The FDA has granted succinic acid with the GRAS status (Generally Recognized as Safe Substance). These results suggested that succinic acid and oxalic acid may endow *Z. rouxii* under salt concentrations with special flavor.

## Conclusion

In summary, we successfully characterized the transcriptomic profiles and the change of main flavor substance of *Z. rouxii* under salt treatment. *Z. rouxii* exhibited a good tolerance toward high concentrations of salt. Analysis of physiological data showed a accumulation of unsaturated fatty acids, succinic acid and oxalic acid. In addition, volatile substances analysis showed that concentrations of 3-methyl-1-butanol and phenylethyl alcohol were significantly increased with the increment of salt concentrations. Transcriptomic analysis showed an up-regulated expression of the genes related to TCA cycle and the linoleic acid synthesis. This study helps to understand the change of main flavor substance of *Z. rouxii* under salt treatment and guide their applications in the high salt liquid state fermentation of the soy sauce.

## Data availability statement

The RNA-seq data presented in the study are deposited in the NCBI/SRA repository (www.ncbi.nlm.nih.gov/sra), accession number: PRJNA837122.

## Author contributions

BH and MA conceived and designed the experiments. BG and RP performed the experiments. GL and YL analyzed the data. RW contributed to reagents, materials, analysis tools, and revised the manuscript. GL and RP wrote the manuscript. All authors contributed to the article and approved the submitted version.

## Supplementary material

Supplementary Figure 1 Proportion of unsaturated and saturated fatty acids content in *Z. rouxii* at different salt concentrations.

Supplementary Figure 2 Relative expression level of six genes encoding crucial enzymes involved in linoleic acid biosynthesis pathway in *Z. rouxii* under different salt concentrations, 0, 6, 12, and 18% represent different salt concentrations, respectively. Gene 10103, 5047, 217, and 5506 are encoding elongase and gene 5111 and 1576 are encoding D12D, separately. The bars represent the average (±SE) of biological repeats. ^ns^, *, **, and *** indicate statistically significant differences between the control and salt-treated groups (the least significant difference test): ns, no significant difference; **p* < 0.05; ***p* < 0.01; ****p* < 0.001.

Supplementary Table 1 qRT-PCR primers used in this study.

Supplementary Table 2 Volatile compounds identified in this study.
