# Supplementary figures and images for: Physiological and transcriptomic analyses revealed the change of main flavor substance of Zygosaccharomyces rouxii under salt treatment
Source: Front Nutr. 2022 Aug 24;9:990380. doi: 10.3389/fnut.2022.990380 (PMC9449518; doi:10.3389/fnut.2022.990380)

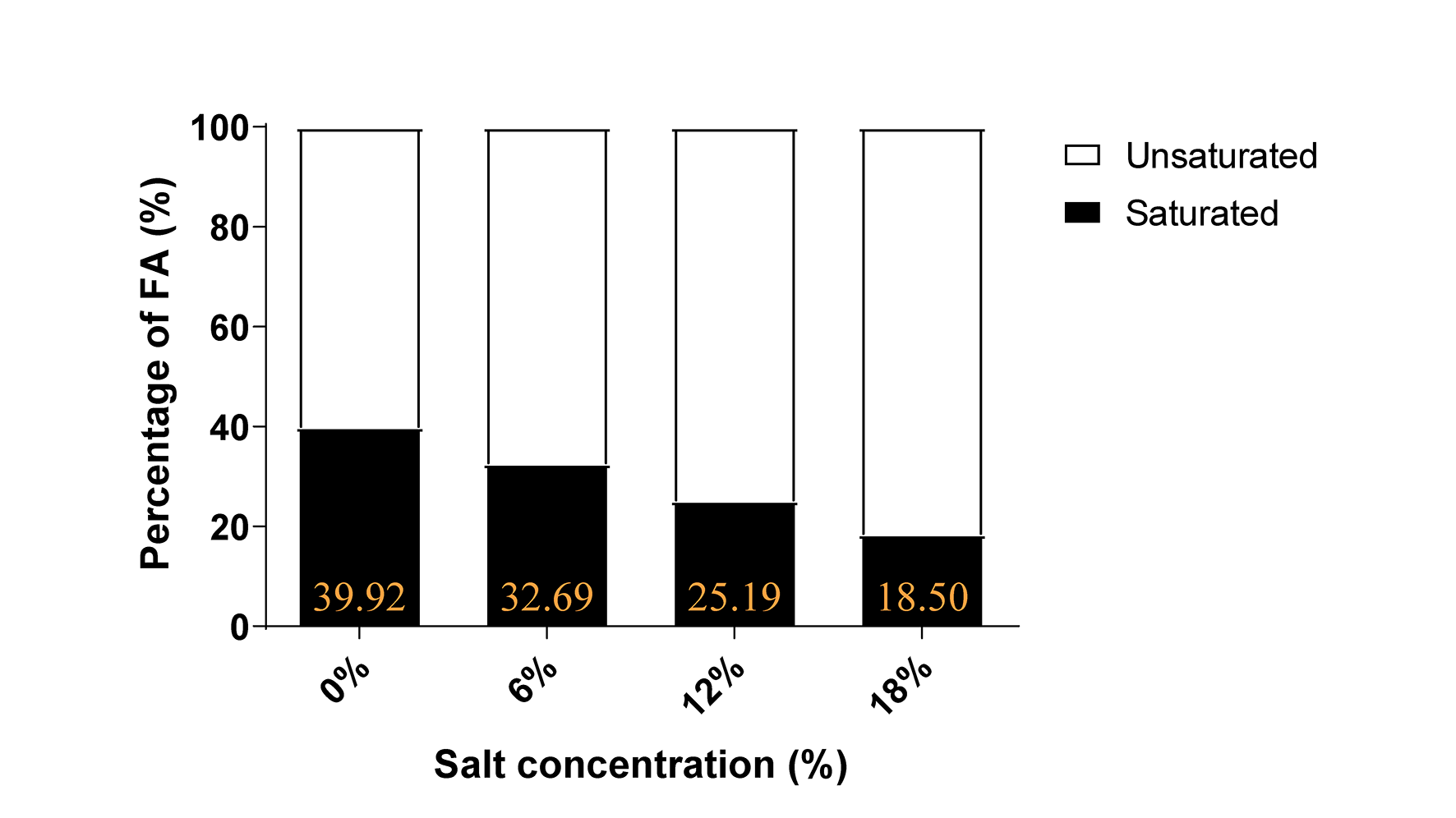

Supplement: Supplementary Figure 1 — Proportion of unsaturated and saturated fatty acids content in Z. rouxii at different salt concentrations. [file Image_1.TIFF]

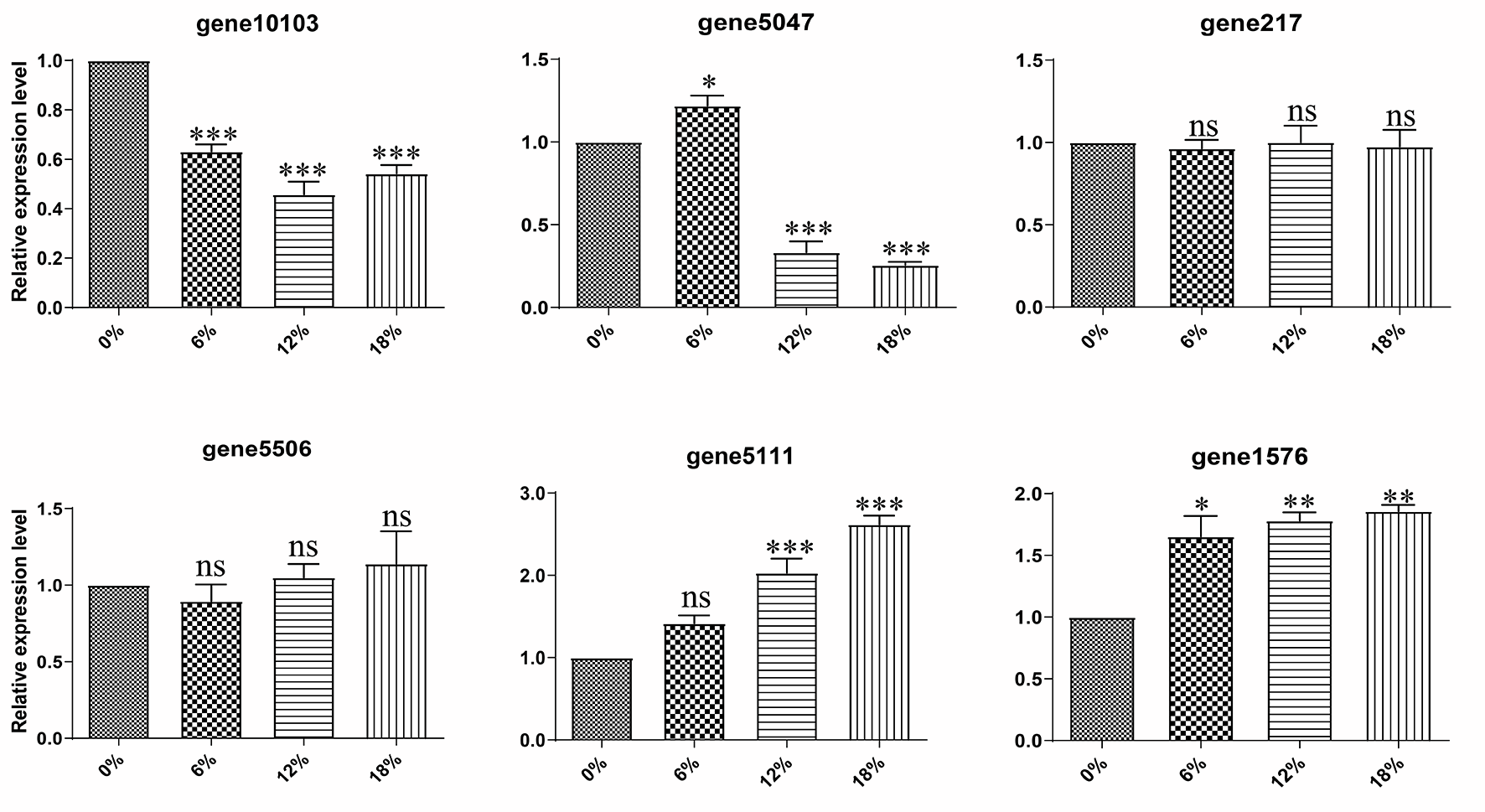

Supplement: Supplementary Figure 2 — Relative expression level of six genes encoding crucial enzymes involved in linoleic acid biosynthesis pathway in Z. rouxii under different salt concentrations, 0, 6, 12, and 18% represent different salt concentrations, respectively. Gene 10103, 5047, 217, and 5506 are encoding elongase and gene 5111 and 1576 are encoding D12D, separately. The bars represent the average (±SE) of biological repeats. ns, *, **, and *** indicate statistically significant differences between the control and salt-treated groups (the least significant difference test): ns, no significant difference; *p < 0.05; **p < 0.01; ***p < 0.001. [file Image_2.TIF]
