# Supplementary material for: Physiological and transcriptomic analyses revealed the change of main flavor substance of Zygosaccharomyces rouxii under salt treatment
Source: Front Nutr. 2022 Aug 24;9:990380. doi: 10.3389/fnut.2022.990380 (PMC9449518; doi:10.3389/fnut.2022.990380)
Supplement: Supplementary Table 1 — qRT-PCR primers used in this study. [file Table_1.DOCX]

**Supplementary Table S1. qRT-PCR Primers used in this study**

| **Primer** | **Sequence (5′-3′)** | **Purpose** |
| --- | --- | --- |
| gene10103-F | GTTGTGCGGTTGTGGAGATA | qRT-PCR of gene10103 |
| gene10103-R | TCCTGTCAGCTAGGGAGTAAA | qRT-PCR of gene10103 |
| gene5047-F | CTGTGCAGTCGTGGAGATATT | qRT-PCR of gene5047 |
| gene5047-R | GGCCACACCAGTTGGATATAG | qRT-PCR of gene5047 |
| gene217-F | GACCGCGATTTCCAAGAATAAC | qRT-PCR of gene217 |
| gene217-R | CAGCGAAGAACTCATCTGAAAC | qRT-PCR of gene217 |
| gene5506-F | ATGGATCACTTTCACCCTCATT | qRT-PCR of gene5506 |
| gene5506-R | CTTCTTCTGAGCCCACAGATAC | qRT-PCR of gene5506 |
| gene5111-F | TTCGGTGGATCTGTTGGATTAG | qRT-PCR of gene5111 |
| gene5111-R | TGTGGATCTCTTTCCGTATCTTG | qRT-PCR of gene5111 |
| gene1576-F | GATCTGCCAGTGTGGGATAAG | qRT-PCR of gene1576 |
| gene1576-R | CTGGGTGCTCGGTCAAATAA | qRT-PCR of gene1576 |
